# Which social determinants of health have the highest impact in community oncology to advance patient care equity and improve health outcomes? A scoping review

**DOI:** 10.1002/cam4.70160

**Published:** 2024-09-06

**Authors:** Kayleigh R. Majercak, Emily F. Gorman, Nicholas J. Robert, Barbara Palmer, Henry Asante Antwi, C. Daniel Mullins

**Affiliations:** ^1^ School of Pharmacy University of Maryland Baltimore Baltimore Maryland USA; ^2^ The PATIENTS Program University of Maryland Baltimore Baltimore Maryland USA; ^3^ Health Sciences and Human Services Library University of Maryland Baltimore Baltimore Maryland USA; ^4^ Ontada Boston Massachusetts USA

**Keywords:** cancer, community oncology, health disparities, health equity, healthcare outcomes, intersectionality, social determinants of health

## Abstract

**Introduction:**

To better understand the SDOH‐health equity landscape within a community oncology setting to answer the research question, “Which SDOH can have the highest impact in community oncology to advance patient care equity and improve health outcomes?”

**Methods:**

Arksey and O'Malley's scoping review framework was used to identify evidence related to SDOH and health equity in community oncology. The study was guided by the “10‐Step Framework for Continuous Patient Engagement” and a Community Advisory Board to assure relevance to patients and community providers. Literature was retrieved from literary databases and oncology organizations' websites. Eligible studies included discussion of SDOH and health equity as outlined by the World Health Organization and Centers for Disease Control and Prevention, respectively, and involved community oncology/cancer care in outpatient settings. Studies were excluded if the SDOH‐health equity relationship was not discussed.

**Results:**

The review resulted in 61 exploratory and 17 confirmatory “intervention” studies addressing the impact of SDOH on health equity in community oncology settings. The most frequently SDOH‐health equity pairs identified were the SDOH categories, *social inclusion and non‐discrimination*, *income and social protection,* and *structural conflict,* all paired with the health equity category, *access to care/treatment*. Confirmatory studies focused on *income and social protection* (SDOH) and *access to care/treatment* (health equity); the SDOH categories, *social inclusion and non‐discrimination* and *health/general literacy–patient,* paired with the health equity category, and *adherence/compliance*.

**Conclusions:**

Literature highlights the SDOH and health equity relationship within the realm of oncology. Most studies on SDOH/health inequities in the community oncology setting are exploratory. There is the need to shift from documentation of cancer inequities to implementing solutions.

## Graphical abstract

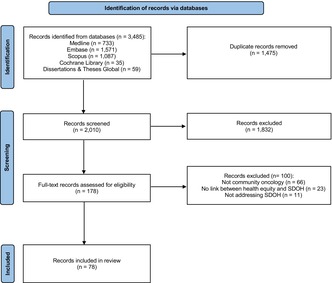

## 1 INTRODUCTION

Despite significant innovations in cancer prevention and treatment, and a longstanding focus on reducing cancer health disparities, unequal health outcomes and access to care remain as current public health challenges. Cancer health disparities are indisputable among marginalized, underserved, and vulnerable populations in the United States.^1^ Specifically, these “adverse differences in cancer burden” affect racial and ethnic minorities, geographically disadvantaged communities, sexual and gender minorities, older adults, socioeconomically disadvantaged, and underinsured individuals.^1^ Increasing evidence consistently demonstrates how an individual's environment and the context of care need to be addressed to achieve better health outcomes and advance health equity.^2^, ^3^, ^4^ Social determinants of health (SDOH) are “the conditions in which people are born, grow, work, live, and age and the wider set of forces and systems shaping the conditions of daily life.”^5^ Individuals living in less affluent areas characterized by suboptimal conditions including but not limited to food insecurity, unemployment, inadequate housing, transportation needs, social exclusion, or lacking opportunities to access healthcare services of decent quality.

SDOH often lend to health inequities for individuals.^2^, ^3^, ^4^ In a 2017 scoping review of SDOH literature within population and public health, Lucyk et al. found health equity ingrained in SDOH.^6^ Health inequities, the “unfair and avoidable differences” are grounded in societal norms as well as political and economic systems.^5^ These factors may be reflected differently across populations, which can give rise to unfavorable health outcomes, especially with costly conditions like cancer. For instance, the cancer mortality rate for individuals residing in rural areas is higher when compared to individuals living in urban areas.^7^ Rural populations are less likely to participate in cancer screenings due to lack of access to cancer screening services or healthcare facilities for follow up for abnormal results, resulting in late‐stage cancer diagnoses. Likewise, older adults, racial/ethnic minorities, and low‐income populations are less likely to receive definitive treatment for their cancer diagnosis.^7^, ^8^ This not only affects the individual, but society as a whole. The estimated total economic burden of cancer in the United States in 2017 was $342.2 billion USD resulting from cancer healthcare costs, lost productivity, and premature death.^9^

Academic medical centers, a type of healthcare facility usually affiliated with a university hospital, are associated with higher cost, and often, these centers receive public funding and provide care for a large proportion of severe and complex cases.^10^ In contrast, community oncology, a practice “not part of a hospital or academic or medical teaching institution” (p. 1), is designed to deliver lower cost, accessible, quality care to individuals diagnosed with cancer.^11^ Nonetheless, community oncology experiences the challenges of the SDOH influence on patient care equity. In a recent cross‐sectional survey study by Zettler et al., oncologists recognized the impact that SDOH (i.e., financial security/lack of insurance and transportation) inflicted upon health outcomes, but, lack of accessible resources (e.g., assistance programs) were identified as a barrier to addressing SDOH.^12^ Interventions such as navigation services in the community oncology setting have been integrated as one way to address patient needs by connecting individuals to resources, yet challenges still exist as health inequities remain.^7^, ^13^, ^14^, ^15^

For community oncology to optimize care for all patients, there is a need to understand the current landscape in terms of what we know about how SDOH impacts cancer care outcomes and how addressing SDOH can lend to high‐quality cancer care in community settings. By doing so, we can inform community oncology care as well as identify where more research is needed. Extensive research has shown the relation between SDOH and health inequities.^5^ Reviews have been conducted highlighting barriers or unmet needs for specific populations, types of cancer, or oncology services.^16^, ^17^, ^18^, ^19^, ^20^, ^21^, ^22^, ^23^ Literature specifically focusing on the intersection of community oncology, SDOH, and health inequities is expanding; however, no scoping review has addressed the question, “Which SDOH can have the highest impact in community oncology to advance patient care equity and improve health outcomes?” This study attempts to fill the knowledge gap by conducting the first scoping review of the literature to better understand the SDOH‐health equity landscape with a community oncology lens. As compared to previous reviews, this review is broader in scope as it extends across various populations, types of cancer, as well as encompasses a wide array of SDOH and their connection to health inequities.

## 2 METHODS

A scoping review was conducted to methodically identify evidence related to social determinants of health (SDOH) and health equity in community oncology. A scoping review “provides a preliminary assessment of the potential size and scope of available research literature. It aims to identify the nature and extent of research evidence” (p. 101).^24^ To differentiate, a scoping review is not a systematic review of the literature, which involves assessing the quality of individual studies. A systematic review did not fit our research purpose, and therefore, we chose the “scoping review” methodology as it is ideal for broad topics instead of narrowly focused topics. Moreover, this review was conducted according to Arksey and O'Malley's scoping review framework: (1) defining the research question, (2) searching the evidence, (3) selecting the evidence, (4) extracting or “charting” the data, and (5) summarizing and reporting the evidence.^25^ A scoping review protocol template was developed to guide the search. To include a patient‐centered component, the “10‐Step Framework for Continuous Patient Engagement” was intertwined with the scoping review process.^26^

A Community Advisory Board (CAB) consisting of patient advisors (three patients and one caregiver), clinicians, researchers, and industry representatives defined the research question to frame the scoping review. The CAB included 17 participants. The research question was finalized during a 3‐h CAB meeting. To emphasize putting patients first, the meeting opened with a “Voice of the Patient” introduction by one of the patient advisors to share the importance of this project based on her experience with cancer. In addition, an “Icebreaker” exercise followed by a series of polling questions and allotted time for reactions and questions created a safe environment for engagement. This environment cultivated a space for all members of the CAB to share their input openly and freely to allow for all voices to be heard. For example, the patient advisors suggested adding “patient care” to the research question to keep the group focused on why we were there and what this project aimed to accomplish. The research question was framed as “Which SDOH can have the highest impact in community oncology to advance patient care equity and improve health outcomes?”

Search strategies were developed by an experienced health sciences librarian in consultation with an advisory committee that included a subset of representatives (*n* = 10) from the CAB: patient advisors (one patient and one caregiver), clinicians, researchers, and industry representatives. The strategies were reviewed by another experienced health sciences librarian prior to implementation. Literature was retrieved from the following databases on April 15, 2022: Medline (Ovid), Embase (Elsevier), Cochrane Library (WileyOnline), Scopus (Elsevier), and Dissertations & Theses Global (ProQuest). Gray literature sources included Trip Database Pro (searched on March 19, 2022) and various oncology‐related websites (searched in October 2022 and searched on February 3, 2023, for the Community Oncology Alliance website). Search strategies combined key terms and subject headings related to SDOH, health equity, and oncology. Full database and gray literature search strategies are available in Appendix A. To ensure a comprehensive search, no search limits or filters were applied.

Eligible studies included discussion of: (1) SDOH (e.g., *income and social protection*), (2) health equity (e.g., *quality of life*), and (3) involved community oncology or cancer care in an outpatient setting (i.e., “not part of a hospital or academic or medical teaching institution”^11^). The categories of SDOH and health equity were outlined by the World Health Organization (WHO) and Centers for Disease Control and Prevention (CDC), respectively.^5^, ^27^ The full list of SDOH and measures of health equity are available in Table 1. Studies involving hospitals, rehabilitation facilities, skilled nursing facilities, or any other inpatient setting were excluded. Studies were also excluded if they did not discuss a relationship or link between social determinants of health and health equity, or if they involved very low‐risk cancers and did not specifically mention treatment by an oncologist.

**TABLE 1:** Categories of social determinants of health (SDOH) and measures of health equity.

SDOH	Health equity
***Income and social protection***	***Rates of disease—prevalence*** ^a^
***Education***	***Rates of disease—incidence*** ^a^
***Unemployment and job insecurity***	***Disability***
***Working life conditions***	***Death***
***Food insecurity***	***Severity of disease***
***Housing, basic amenities, and the environment***	***Quality of life***
***Early childhood development***	***Access to care/treatment*** ^a^
***Social inclusion and non‐discrimination***	***Overall survival*** ^a^
***Structural conflict***	Progression‐free survival^a^
***Access to affordable health services of decent quality***	Disease‐free survival^a^
Health/general literacy—patient^a^	Time to progression^a^
Patient—provider communication^a^	Time to next therapy^a^
Preparing for care^a^	Duration of therapy^a^
	Adherence/compliance^a^
	Multiple primaries^a^
	Recurrence of cancer^a^
	Access to precision medicine^a^

*Note*: SDOH categories bolded and italicized are pre‐defined based on the World Health Organization's list of SDOH.^5^ Health equity categories bolded and italicized are based on Centers for Disease Control and Prevention's “Health Equity” summary examples.^27^

^a^ Categories added or revised using Community Advisory Board (CAB) input.

It is important to note, CDC does not have a designated list of health equity measures, but examples were described in their “Health Equity” summary.^27^ Additional SDOH and health equity categories were included based on input from the CAB. For example, input from patient advisors highlighted how preparing for care was important. Part of the issue stemmed from providers and their teams not providing enough time to explain the details of the recommended treatments such as side effects, or alternative therapies. Furthermore, “patients will not go for a second opinion, [patients] just believe, …doesn't ask questions.” Therefore, communication regarding cancer care should be clear. As a result, *preparing for care* as well as *health literacy* and *general literacy* categories, both at the patient and provider level, were added to the WHO list of SDOH. Other CAB members provided additional categories for health equity measures or suggested existing categories be revised. For example, *access to precision medicine* was added. CDC's original terms such as “length of life” were revised to *overall survival*, “rates of disease” adapted to include both prevalence and incidence separately, and “access to treatment” was expanded to include *access to care/treatment*.

The online Covidence software was used to remove duplicates and facilitate screening of the materials retrieved from the literature databases.^28^ Titles and abstracts for each record were independently screened by two reviewers, and conflicts were resolved through discussion and consensus. The full texts of relevant records were retrieved and independently screened by two reviewers, with conflicts resolved through discussion and consensus. A third reviewer served as a tiebreaker if consensus was unable to be reached. If records were excluded during the full‐text review, a rationale for exclusion was provided following an exclusion hierarchy: (1) Does not contain any SDOH, (2) Does not meet our definition of community oncology based on inclusion/exclusion criteria, or (3) A link between the SDOH and the measure of health equity was not provided. Website records were screened outside of Covidence using an Excel spreadsheet. One reviewer screened each web document for inclusion or exclusion and a second reviewer conducted a quality check. Similar to the review of literature databases, a third reviewer served as a tiebreaker for any discrepancies that were unable to be resolved through discussion.

The following data were extracted from the included studies: year of study, geographic location, setting, services provided by the clinic, study design, data source(s), participant characteristics, population characteristics (e.g. education, employment status and income), method of recruiting participants, type of SDOH, measure of health equity, cancer type and stage, prevalence of anxiety and/or depression (if reported), oncology intervention, and measures of trust and/or support (if reported). Data were extracted primarily from the methods section as well as the descriptive study population table and main study findings presented in the results. For studies involving qualitative research, quotations from interviews, focus groups, or open‐ended response surveys supporting the main study findings were reviewed when applicable. Data extraction was conducted in Covidence by two independent reviewers. Data were exported as a Microsoft Excel file and imported into SAS 9.4 (SAS Institute Inc., Cary, NC, USA) software to build the analytical file and conduct analyses. Descriptive statistics were performed for the analysis using the attributes extracted via frequency counts.

## 3 RESULTS

**FIGURE 1:**
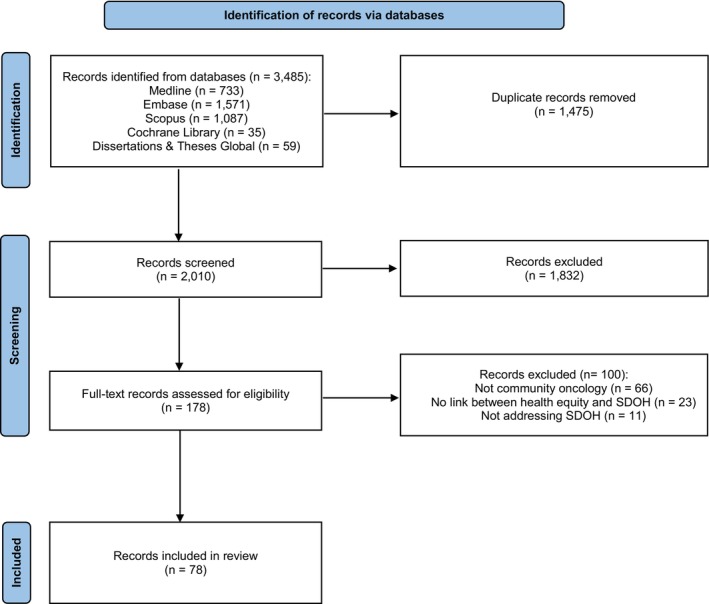
PRISMA diagram of selection process for included records in scoping review.

Of the *n* = 3485 records identified from databases, *n* = 1475 duplicate records were removed leaving *n* = 2010 database records for abstract review. During abstract review, *n* = 1832 records were eliminated and the remaining *n* = 178 database records were screened via full‐text review. The *n* = 100 records that did not focus on community oncology (*n* = 66), did not address SDOH (*n* = 11), or did not demonstrate a link (i.e., measure the association) between the measure of health equity and SDOH (*n* = 23) were eliminated. As a result, *n* = 78 records met the inclusion criteria for analysis (Figure 1). The percentage of agreement between reviewers for abstract screening was 80.1%.

The scoping review identified peer‐reviewed literature published between the years 1996 to 2022, with most of the literature published recently in the year 2021 (*n* = 20). Research efforts were largely exploratory studies (78.2%) in contrast to confirmatory studies (21.8%). Exploratory studies are defined as studies dedicated to identifying SDOH issues that impact health equity, for example, qualitative research and regression models identifying predictors of health inequities. Confirmatory studies refer to interventions addressing SDOH issues and assessing the impact on health equity. For example, a study assessing the impact of navigation services on addressing patient needs or the impact of an educational program on cancer screening behavior. Confirmatory studies glean knowledge from the exploratory studies by leveraging the documented cancer disparities to act and implement health equity solutions. Moreover, the type of study design as described in Table 2 was primarily classified as qualitative research (30.8%), literature review (20.5%), and cross‐sectional study (14.1%). For instance, interviews were conducted in communities to explore barriers and facilitators related to accessing health care or treatment. Literature focused primarily on Black/African American populations (*n* = 45) and similarly included both female and males in research (Table 3). In addition to inequities related to race/ethnicity and gender, inequities related to age, sexual minority populations, and geographic location were studied. Breast cancer was the predominant type of cancer included in studies and identified in approximately 42% of the studies (Table S1).

**TABLE 2:** Study design characteristics (*n* = 78).

Study design	Frequency
Case report	1
Cohort study	2
Cross‐sectional study	11
Descriptive study	6
Ecological studies	1
Literature review	16
Qualitative research	24
Randomized controlled trial	5
Scoping review	3
Systematic mapping review, Retrospective cohort, Descriptive Study, Qualitative research^a^	1
Systematic review	6
Text and opinion/Commentary	2

^a^ Denotes one record containing multiple research studies.

**TABLE 3:** Race, ethnicity, and sex characteristics reported (*n* = 78).

	Frequency
Race	
American Indian or Alaska Native	6
Asian	13
Black/African American	45
Native Hawaiian or Other Pacific Islander	4
White	29
Other Race	16
Undisclosed	30
Ethnicity	
No, Not of Hispanic, Latino, or Spanish Origin	11
Yes, of Hispanic, Latino, or Spanish Origin	29
Undisclosed	38
Sex	
Female	44
Intersex	1
Male	31
Undisclosed	31

The type of SDOH and measures of health equity identified from the included database records varied. Research efforts and initiatives concentrated on *social inclusion and non‐discrimination* across several measures of health equity related to race/ethnicity, age, and sexual orientation. Likewise, *access to care/treatment* was a health equity measure of interest. The top three most widely studied pairs of SDOH concepts and measures of health equity in literature were as follows: *social inclusion and non‐discrimination* and the impact on *access to care*/*treatment* (*n* = 38); *income and social protection* and *access to care*/*treatment* (*n* = 30); and *structural conflict* and *access to care/treatment* (*n* = 14) (Table 4). Based upon the inclusion/exclusion criteria, literature was not available for all the categories of SDOH (i.e., *early childhood development*) and measures of health equity (i.e., *disability*) outlined in Table 1.

**TABLE 4:** Frequency: Type of Social Determinants of Health (SDOH) and measures of health equity reported (*n* = 78).

		Measures of Health Equity									
		OS	TTNT	Adh/comp	Rates dx‐inc	Rates dx‐prev	Death	Sev of dx	ATC/Tx	ATPM	QOL
Social Determinants of Health (SDOH)	Income & soc prtctn	1		7			3	1	30	5	5
	Education	1		7		1		1	10	2	3
	Unemp & job sec			1							1
	Work life conditions				1						
	Food insecurity					2			1		
	House, amenities, environ		1	4	1	4	1	1	13	2	2
	Soc incl & non‐discrim	1	4	13	3		1	1	38	7	5
	Structural conflict	1		3					14		
	Access afford hlth srv	1	2	4			1	2	7	1	2
	Preparing for care			1					8		1
	Hlth/Gen lit—pat	1		12					8	3	
	Pat‐provider comm			1					14	1	

*Note*: Cells are shaded from darkest to lightest to indicate the SDOH‐health equity pairs that are most frequently reported in literature.

Abbreviations: *SDOH*: Access afford hlth srv, access to affordable health services of decent quality; Hlth/gen lit—pat, health/general literacy—patient; House, amenities, environ, Housing, basic amenities, and the environment; Income & soc prtctn, income and social protection; Pat‐provider comm, patient–provider communication; Soc incl & non‐discrim, social inclusion and non‐discrimination; Unemp & job sec, unemployment and job security; work life cond, working life conditions. *Measures of Health Equity*: Adh/comp, adherence/compliance; ATC/Tx, access to care/treatment; OS, overall survival; QOL, quality of life; Rates dx‐inc, rates of disease‐incidence; Rates dx‐prev, rates of disease‐prevalence; Sev of dx, severity of disease; TTNT, time to next therapy.

Of the confirmatory studies identified (*n* = 17), several confirmatory studies reported on multiple SDOH‐health equity pairs (Table 5).^22^, ^29^, ^30^, ^31^, ^32^, ^33^, ^34^, ^35^, ^36^, ^37^, ^38^, ^39^, ^40^, ^41^, ^42^, ^43^, ^44^ Areas of focus for these studies consisted of *social inclusion and non*‐*discrimination* and *adherence*/*compliance* (*n* = 5), *health*/*general literacy*–*patient* and *adherence*/*compliance* (*n* = 5), *income and social protection* and *access to care*/*treatment* (*n* = 5), and *social inclusion and non‐discrimination* and *access to care*/*treatment* (*n* = 4). Again, the areas of focus may not be mutually exclusive as a study could report on multiple pairs. The majority of the SDOH‐health equity pairs contained in studies were deemed as “informational.” Studies included qualitative studies, literature reviews (e.g., quantitative results not specified), or “in‐progress” studies (e.g., randomized controlled trial). For the confirmatory studies with quantitative results (*n* = 9), results generally demonstrated a positive impact. For example, telehealth may be a viable option to improve access to care and reduce transportation costs for individuals residing in rural areas. However, educational interventions were shown to have mixed results. No studies reported interventions being associated with a negative impact. An extension of Table 5 is available as Table S2 for a detailed description of results.

**TABLE 5:** Confirmatory studies addressing SDOH and assessing the impact on health equity (*n* = 17).

Author (year)	Study design	Cancer type	Geography/population characteristics	SDOH/health equity pair		Impact
Adams (1996)^29^	Cross sec	CRC	Texas, Urban; Black/African American, Female and Male, Aged 35–66 years old	Hlth/gen lit‐pat	Adh/comp	NC
Carthon (2021)^30^	Sys Rev	PC	Undisclosed geography; Black/African American and White, Male	Education	Adh/comp	+
				Education	ATC/Tx	+
Chavarria (2021)^31^	Qual; RCT	BC, CRC	Undisclosed geography; Black/African American, White, and Other including Hispanic, Latino, or Spanish origin, Female and Male	Hlth/gen lit‐pat	ATC/Tx	Info
				Soc incl & non‐discrim	Adh/comp	NC
Gil (2016)^32^	Descrip	ALL, MEL, MB	Undisclosed geography; Hispanic, Latino, or Spanish origin	Hlth/gen lit‐pat	Adh/comp	Info
				Soc incl & non‐discrim	Adh/comp	Info
				Soc incl & non‐discrim	ATPM	Info
Holle (2020)^33^	Descrip	CRC	Connecticut, Urban; Black/African American and White including Hispanic, Latino, or Spanish origin, Minimum age 45 years old	Access afford hlth srv	Adh/comp	Info
				Hlth/gen lit‐pat	Adh/comp	Info
				Income & soc prtctn	Adh/comp	Info
				Prov comm & cultrl comp	ATC/Tx	Info
Kim (2016)^22^	Sys Rev	CC, BC, CRC, OC	United States, Canada, India, Pakistan, Taiwan; Asian, Black/African American, White including Hispanic, Latino, or Spanish origin, Female and Male, Aged 32–71 years old	Income & soc prtctn	ATC/Tx	Info
				Soc incl & non‐discrim	ATC/Tx	Info
Meade (2020)^34^	Lit Rev	U	Undisclosed geography and population characteristics	Prov comm & cultrl comp	Adh/comp	Info
				Prov comm & cultrl comp	ATC/Tx	Info
				Soc incl & non‐discrim	Adh/ comp	Info
				Soc incl & non‐discrim	ATC/ Tx	Info
				Soc incl & non‐discrim	QOL	Info
Okoro (2020)^35^	Qual	U	Minnesota, Urban; Black/African American and Other, Female and Male, Aged 18–86 years old	Education	Adh/ comp	Info
				Education	ATC/Tx	Info
				Education	QOL	Info
				Hlth/gen lit‐pat	ATC/Tx	Info
				House, amenities, environ	QOL	Info
Otero^a^ (2022)^36^	Qual	PC, PaC	Undisclosed geography; Black/African American and Other including Hispanic, Latino, or Spanish origin	Hlth/gen lit‐pat	Adh/comp	Info
				House, amenities, environ	ATC/Tx	Info
				Education	Adh/comp	Info
				Preparing for care	ATC/Tx	Info
				Soc incl & non‐discrim	Adh/comp	Info
Patel (2020)^37^	RCT	LC, BC, GI, GU, HEM, U	New Jersey, Illinois, Urban; Asian, Black/African American, Native Hawaiian or Other Pacific Islander, White, Female and Male, Aged at least 18 years old	Income & soc prtctn	ATC/Tx	Info
				Soc incl & non‐discrim	ATC/Tx	Info
				Access afford hlth srv	QOL	Info
Patel^a^ (2021)^38^	RCT	BC, LC, U	New Jersey; Black/African American, White, and Other, Female and Male	Income & soc prtctn	ATC/Tx	+
				Income & soc prtctn	QOL	+
				Preparing for care	ATC/Tx	+
				Preparing for care	QOL	+
				Soc incl & non‐discrim	QOL	+
Percac‐Lima (2014)^39^	Cohort	BC	Massachusetts, Urban; Asian, Black/African American, White, and Other including Hispanic, Latino, or Spanish origin, Female	Access afford hlth srv	TTNT	+
Rariy^a^ (2021)^40^	Cross sec	U	Rural; Undisclosed population characteristics	Access afford hlth srv	TTNT	+
				House, amenities, environ	ATC/Tx	+
				House, amenities, environ	TTNT	+
Reynolds^a^ (2020)^41^	Cohort	U	Undisclosed geography; Asian, Black/African American, and Other including Hispanic, Latino, or Spanish origin	Soc incl & non‐discrim	ATPM	+
Smith (2016)^42^	Descrip	BC	Illinois, Urban; Black/African American, Female	Hlth/gen lit‐pat	ATC/Tx	Info
				Hlth/gen lit‐pat	Adh/comp	Info
				Soc incl & non‐discrim	Adh/comp	Info
Strom^a^ (2017)^43^	Descrip	BC, GI, HEM, Thorac	North Carolina; Black/African American and Other including Hispanic, Latino, or Spanish origin, Female and Male	Income & soc prtctn	ATC/Tx	+
Thompson (2018)^44^	Lit Rev	BC	Illinois, Ohio, Washington; Black/African American and White including Hispanic, Latino, or Spanish origin, Female	Access afford hlth srv	Death	Info
				Education	ATC/Tx	+
				Income &soc prtctn	ATC/Tx	+
				House, amenities, environ	ATC/Tx	+
				Soc incl & non‐discrim	ATC/Tx	+

*Note*: Results are based upon confirmatory studies (i.e., interventions) addressing social determinants of health (SDOH) and assessing the impact on health equity measures. Results from exploratory studies identifying SDOH and health equity issues were not included.

Abbreviations: *Study Design*: Cross sec, cross‐sectional; Descrip, descriptive; Lit Rev, literature review; RCT, randomized controlled trial; Sys Rev, systematic review; Qual, qualitative research. *Type of Cancer*: ALL, acute lymphoblastic leukemia; BC, breast cancer; CC, cervical cancer; CRC, colorectal cancer; GI, gastrointestinal; GU, genitourinary; HEM, hematologic; LC, lung cancer; MB, medulloblastoma; MEL, melanoma; OC, oral cancer; PaC, pancreatic cancer; PC, prostate cancer; Thorac, thoracic; U, undisclosed. *SDOH*: Access afford hlth srv, access to affordable health services of decent quality; Hlth/gen lit‐pat, health/general literacy patient; House, amenities, environ, housing, basic amenities and the environment; Income & soc prtctn, income and social protection; Pat‐provider comm, pat‐provider communication; Prep for care, preparing for care; Soc incl & non‐discrim, social inclusion and non‐discrimination. *Health Equity*: Adh/comp, adherence/compliance; ATC/Tx, access to care/treatment; ATPM, access to precision medicine; QOL, quality of life; *Impact*: +, positive; Info, informational; NC, no change.

^a^ Abstract only.

The scoping review of the gray literature (i.e., websites) included a total of *n* = 24 records. Compared with the scoping review of literature via databases, the review process for websites did not include abstract screening (Figure S1). These results align with the findings from the peer‐reviewed literature. Both search strategies identified similar top pairs of SDOH and measures of health equity; however, the ranked frequency of pairs identified may differ slightly. For example, *social inclusion and non‐discrimination* and *income and social protection* demonstrated a reversed ranking for the top two positions among the most frequently reported SDOH for peer‐reviewed literature and gray literature, respectively. Both SDOH concepts were paired with *access to care*/*treatment* as the measure of health equity. Additionally, the SDOH and health equity pair, *access to affordable health services of decent quality* and *access to care/treatment*, was frequently reported in the gray literature whereas this SDOH‐health equity pair was not as frequently reported in the peer‐reviewed literature. Interestingly, in contrast to the peer‐reviewed literature, gray literature existed for two measures of health equity: *duration of therapy* and *recurrence of cancer* identified from one record.

## 4 DISCUSSION

This review resulted in 61 exploratory studies identifying SDOH impacting health equity and 17 confirmatory studies addressing SDOH and assessing the impact on health equity. Overall, the most frequently SDOH‐health equity pair identified in literature was *social inclusion and non‐discrimination* and *access to care*/*treatment*. Predominately, confirmatory studies have focused on addressing *access to care*/*treatment* via *social inclusion and non*‐*discrimination* and *income and social protection* as well as addressing *adherence*/*compliance* via *social inclusion and non‐discrimination* and *health*/*general literacy*–*patient*. It is important to note research is limited regarding confirmatory studies, especially with quantitative results.

The topic of understanding cancer in minority and underserved populations is not new. In his 1997 statement on the role of the National Cancer Institute (NCI) in addressing cancer in minority and underserved populations, Dr. Richard Klausner expressed the need for “good data.”^45^ “We must collect fuller and more informative sets of data …so that they allow us to identify the burden of cancer in groups and to identify the groupings that experience different burdens of cancer” (p. 1703).^45^ Similarly, in response to the request by the U.S. Congress in 1997 for a review of the National Institutes for Health's research for minorities and underserved populations, the Institute of Medicine (IOM) developed and charged a committee for this request.^46^ The IOM committee found the Surveillance, Epidemiology, and End Results (SEER) program to be lacking critical information. “It lacks the necessary database concerning the disproportionate cancer incidence, mortality, and survival rates among ethnic minorities and medically underserved groups that would permit NCI to develop and evaluate effective cancer control strategies for these populations” (p. 9).^46^ Likewise, recommendations from a 2010 C‐Change report on the societal and economic impact of cancer health inequities included the need for better data.^47^ It is encouraging to see that our results indicate efforts to better understand health inequities via exploratory studies.

Contrary to expectations, the review of websites resulted in the identification of two measures of health equity, that is, *duration of therapy* and *recurrence of cancer*, that did not appear in the review of literature from databases. It is important to bear in mind the possibility of publication bias with peer‐reviewed studies, that is, publishing studies that demonstrate a favorable statistically significant difference in results. It is well known there are fewer studies published showing interventions that did not work well in clinical research.^48^ In health services research, it is unknown to what degree publication bias exists.^49^ We observed a higher frequency of favorable results for confirmatory studies and two examples of confirmatory studies that indicated no change between comparison groups. These two confirmatory studies aimed to promote screening/testing for cancer, which is similar to the aims of a few interventions that reported favorable findings. None of the studies reported negative findings. It is encouraged for researchers to publish both favorable and non‐favorable results as the combination of these findings may allow others to learn from to understand what worked or did not work well and therefore, tailor their interventions appropriately.

Our findings are in accordance with the results of the scoping review on SDOH by Lucyk and McLaren with regard to the dominance of downstream‐focused interventions in contrast to upstream‐focused interventions.^6^ Downstream interventions refer to addressing “effects of causes” whereas upstream interventions address “causes‐of‐the‐causes.”^6^ Structural barriers create SDOH challenges and furthermore, the challenges to addressing them. Perhaps the most interesting observation was how studies may include more than one SDOH or measure of equity. This highlights the complexity of SDOH and how they may intersect with one another.^50^ Our research demonstrated *social inclusion and non*‐*discrimination* and *access to care*/*treatment* as an area of focus. *Access to care/treatment* may exist on the causal pathway influencing several downstream outcomes such as overall survival and severity of disease. In the context of community oncology, care at the patient level can be optimized by equipping clinicians with clinical tools to make a “SDOH diagnosis” and connect patients to available support resources.^51^, ^52^ In addition, at the practice‐level, clinician training programs related to cultural communication, implicit biases as well as SDOH awareness and competency can improve patient–provider interactions improving both care and outcomes. The use of best practices can be documented for others to glean and learn from. This will help shift from the documentation of inequities to implementing solutions as interventions are currently limited.

With respect to the original list of SDOH and measures of health equity, we found that we needed to revise the original *general*/*health literacy*–*provider* category to *patient*–*provider communication*. During the review process, we realized this category encompasses more than provider bedside manner and delivering condition and treatment information in language that could be easily understood by patients. The interaction and encounters between provider and patient may be impacted by implicit biases (i.e., attitudes or beliefs that unconsciously impact our decisions).^53^ As a result, this may negatively affect care and access to high‐quality healthcare and treatment. For example, in their review Maina et al. found that provider bias is associated with poorer patient–provider interactions such as higher verbal dominance, less patient‐centeredness, supportive communication, and interpersonal treatment provoking lower patient satisfaction and trust in suggested treatment.^54^

This study has several strengths. A scoping review was conducted to systematically identify evidence related to social determinants of health (SDOH) and health equity in community oncology thereby increasing the quality of our study. Our scoping review may be considered novel, as a patient‐centered component was intertwined with the scoping review process.^26^ Likewise, our study employed a multi‐stakeholder approach. A community advisory board (CAB) was continuously involved throughout the process. The CAB co‐developed the search strategy which included key terms, SDOH and health equity categories, and inclusion and exclusion criteria for the scoping review protocol to ensure the search was extensive. The School of Pharmacy librarian was included among the research team expertise and conducted a comprehensive database search using multiple databases. A team of reviewers performed screening for unbiased article screening (i.e., double‐screened). Weekly team meetings were held to discuss screening and data abstraction thereby cultivating group discussion and ensuring alignment on inclusion/exclusion criteria and that a consistent approach was used for data abstraction.

It is important to note that this study has several limitations. First, the review is not a systematic review of the literature. As mentioned above, a systematic review did not fit our research purpose as our study focused on understanding the current SDOH‐health equity landscape in terms of broad topics instead of narrowly focused topics. Systematic reviews require grading the quality of evidence, whereas we wanted to understand the “scope” of available literature, and therefore, we did not critically assess the included studies. In addition, there may be keywords missing in our search strategy as others may utilize terms that are not as commonly used. Specifically with SDOH, terms may be missing due to the sizeable and expanding list.^55^ We believe this did not have a significant impact on our search strategy as our broad list of conventional terms would retrieve the majority of the literature. As we limited our scope to community oncology, literature may be missing regarding research conducted external to the field of community oncology that may be impactful. For example, payers implementing interventions to address food insecurity, transportation, and housing barriers.^56^, ^57^ Andermann published a framework to help healthcare professionals take action on SDOH by outlining barriers and facilitators with examples used in practice.^51^ Similarly, Bloch and Rozmovits review social interventions and discuss their feasibility in practice (e.g., incorporating specialists/navigators within the team, developing partnerships with community organizations, or if resources are constrained, maintaining a list of resources for patient referral regarding income security, legal, literacy, employment needs).^52^ Nonetheless, our findings (e.g., *social inclusion and non*‐*discrimination* and the impact on *access to care*/*treatment*) may be generalizable to other healthcare settings and conditions beyond oncology, for example, chronic conditions such as asthma. According to the American Lung Association, populations residing in low‐income areas are at a higher risk of developing asthma due to SDOH (i.e., air pollution and structural racism). These areas lack resources such as affordable health services of decent quality thereby increasing rates of asthma severity as individuals are unable to manage their condition.^58^

Compared to other reviews that traditionally search databases, our review included searching websites, which may contain gray literature. The gray literature may not be as methodologically rigorous compared to peer‐reviewed research. However, searching websites did enable us to find additional peer‐reviewed articles or conference abstracts not found via databases, ultimately adding to the review. Moreover, having two platforms to identify literature allowed for the comparison of results, if results were aligned or whether the websites were a source of new information that added to the database findings. Overall, we found the website results to be aligned with the database results. Lastly, the review includes literature that is largely U.S. focused, and therefore, the results may not be applicable to global systems.

## 5 CONCLUSIONS

Currently, there is a strong interest in SDOH in the context of routine healthcare and making SDOH available for research purposes. A plethora of published literature highlights the relationship between SDOH and health equity related to oncology. Most studies on SDOH and health inequities in the community oncology setting are exploratory. There is the need to shift the interest from documentation of cancer inequities to implementing health equity solutions.

Despite the limited availability of published confirmatory research, the existing literature provides evidence of a few SDOH related to *access to care*/*treatment* and *adherence*/*compliance*. With the increasing interest in SDOH and health equity, there has been a proliferation of research articles in recent years and this trend is expected to continue. In a few years, the results may need to be updated as new trends and evidence of SDOH in community oncology emerge and as efforts continue for the development of solutions to advance high‐quality cancer care and health equity.

## AUTHOR CONTRIBUTIONS

**Kayleigh R. Majercak:** Conceptualization (equal); data curation (equal); formal analysis (lead); methodology (equal); project administration (equal); writing – original draft (lead). **Emily F. Gorman:** Conceptualization (equal); data curation (equal); methodology (equal); writing – original draft (supporting). **Nicholas J. Robert:** Conceptualization (equal); supervision (equal). **Barbara Palmer:** Conceptualization (equal). **Henry Asante Antwi:** Conceptualization (equal); data curation (equal); project administration (equal). **C. Daniel Mullins:** Conceptualization (equal); methodology (equal); supervision (equal).

## FUNDING INFORMATION

This study received funding from Ontada, LLC.

## PRECIS

Currently, there is a strong interest in SDOH in the context of routine healthcare and making SDOH available for research purposes. There is the need to shift the interest from documentation of cancer disparities to implementing health equity solutions.

## Supporting information


**Appendix A**—Search Strategies.

**Figure S1.** PRISMA diagram of selection process for included website records in scoping review.

**Table S1.** Cancer Types Reported (*n* = 78).

**Table S2.** Extended Results: Confirmatory Studies Addressing SDOH and Assessing the Impact on Health Equity (*n* = 17).

## Data Availability

The data that support the findings of this study are available from the corresponding author upon reasonable request.
