# Supplementary material for: Which social determinants of health have the highest impact in community oncology to advance patient care equity and improve health outcomes? A scoping review
Source: Cancer Med. 2024 Sep 6;13(17):e70160. doi: 10.1002/cam4.70160 (PMC11378356; doi:10.1002/cam4.70160)
Supplement: Supplementary file 1 — Appendix A—Search Strategies. [file CAM4-13-e70160-s003.docx]

**Appendix A – Search Strategies**

Search strategies developed by Emily F. Gorman, MLIS and reviewed by Andrea G. Shipper, MSLIS

Medline, Embase, Scopus, Cochrane Library, and Dissertations & Theses Global were searched on 15 April 2022; Trip Database Pro was searched on 19 March 2022; oncology websites were searched October 2022 and February 2023.

Filters / Limits: none

**MEDLINE and Epub Ahead of Print, In-Process, In-Data-Review & Other Non-Indexed Citations and Daily (Ovid, 1946 – April 14, 2022) – 733** references retrieved on 15 April 2022

Multi-line search run in the Advanced Search interface:

1. exp social determinants of health/ or exp health services accessibility/ or exp insurance, health/ or exp health literacy/ or exp educational status/ or exp social participation/ or exp community participation/ or exp social inclusion/ or exp social integration/ or exp social cohesion/ or exp social isolation/ or exp loneliness/ or exp social support/ or exp social networking/ or exp social discrimination/ or exp workplace/ or exp prisoners/ or exp economic stability/ or exp income/ or exp economics/ or exp social class/ or exp socioeconomic factors/ or exp public policy/ or exp social environment/ or exp poverty/ or exp employment/ or exp food security/ or exp food supply/ or exp housing/ or exp neighborhood characteristics/ or exp residence characteristics/ or exp crime/ or exp violence/ or exp transportation/ or exp air pollution/ or exp water quality/ or exp access to healthy foods/ or exp food deserts/ or exp communication barriers/ or exp child development/ or exp culture/ or exp stress, physiological/ or exp stress, psychological/ or exp exercise/ or exp parks, recreational/
2. (social determin* or social factor* or social health determinant* or sdoh or health structural determinant* or ((healthcare or care or health services) adj2 access*) or ((health or healthcare or health care or medical or sickness) adj (insurance or benefit*)) or health literacy or ehealth literacy or education level* or level of education or educational attainment or educational achievement or ((civic or social or community or public) adj (participation or engagement or integration or inclusion or involvement)) or sense of community or sense of belonging or isolation or social exclusion or loneliness or social support* or social network* or discrimination or nondiscrimination or non discrimination or racism or homophobia or biphobia or transphobia or workplace condition* or workplace environment or incarceration or imprisonment or prisoner* or economic stability or income or cost of living or socioeconomic* or socio-economic* or social economic status or (social adj (status or achievement or condition* or protection or polic* or environment or connectedness or cohesion or context*)) or public polic* or government* polic* or poverty or employment or unemployment or occupational status or job security or job insecurity or food security or food insecurity or housing or lodging* or living arrangement* or transportation or air quality or air pollution or water quality or healthy food* or food desert* or neighborhood* or neighbourhood* or physical environment or crime or violence or language barrier* or communication barrier* or driver* of health or doh or childhood development or early life development or structural conflict* or culture or stress or physical activit* or exercise or parks or green space* or walkability or walk friendly or walking friendly or playground*).ti,ab,kw.
3. 1 or 2
4. exp health equity/
5. (((equit* or inequit* or equal* or inequal* or unequal*) adj3 (health* or care)) or equitabl* or inequitabl*).ti,ab,kw.
6. 4 or 5
7. exp medical oncology/ or exp antineoplastic protocols/ or exp combined modality therapy/ or exp chemoradiotherapy/ or exp chemotherapy, adjuvant/ or exp consolidation chemotherapy/ or exp electrochemotherapy/ or exp induction chemotherapy/ or exp maintenance chemotherapy/ or exp photochemotherapy/ or exp radiotherapy/ or exp remission induction/ or immunotherapy/
8. (oncolog* or cancerology or ((cancer or tumor* or tumour*) adj3 (care or treat* or therap* or intervention*)) or radiotherap* or chemotherap* or immunotherap*).ti,ab,kw.
9. 7 or 8
10. 3 and 6 and 9

**Embase (Elsevier) – 1571** references retrieved on 15 April 2022

One-line search run in the Quick search tab of the Embase.com interface:

('social determinants of health'/exp OR 'health care access'/exp OR 'health insurance'/exp OR 'health literacy'/exp OR 'educational attainment'/exp OR 'educational status'/exp OR 'civic engagement'/exp OR 'community engagement'/exp OR 'community participation'/exp OR 'social participation'/exp OR 'social inclusion'/exp OR 'integration'/exp OR 'sense of community'/exp OR 'sense of belonging'/exp OR 'isolation'/exp OR 'loneliness'/exp OR 'social support'/exp OR 'social network'/exp OR 'discrimination against sexual and gender minorities'/exp OR 'racism'/exp OR 'workplace'/exp OR 'incarceration'/exp OR 'prisoner'/exp OR 'economic status'/exp OR 'income'/exp OR 'financial security'/exp OR 'cost of living'/exp OR 'socioeconomics'/exp OR 'social status'/exp OR 'social protection'/exp OR 'social environment'/exp OR 'social connectedness'/exp OR 'public policy'/exp OR 'poverty'/exp OR 'employment'/exp OR 'unemployment'/exp OR 'food security'/exp OR 'housing'/exp OR 'neighborhood'/exp OR 'crime'/exp OR 'violence'/exp OR 'physical environment'/exp OR 'traffic and transport'/exp OR 'air quality'/exp OR 'air pollution'/exp OR 'water quality'/exp OR 'healthy food'/exp OR 'food desert'/exp OR 'communication barrier'/exp OR 'child development'/exp OR 'cultural anthropology'/exp OR 'physiological stress'/exp OR 'physical activity'/exp OR 'exercise'/exp OR 'recreational park'/exp OR 'walkability'/exp OR 'playground'/exp OR 'social determin*':ti,ab,kw OR 'social factor*':ti,ab,kw OR 'social health determinant*':ti,ab,kw OR sdoh:ti,ab,kw OR 'health structural determinant*':ti,ab,kw OR (((healthcare OR 'care' OR 'health services') NEAR/2 access*):ti,ab,kw) OR (((health OR healthcare OR 'health care' OR medical OR sickness) NEXT/1 (insurance OR benefit*)):ti,ab,kw) OR 'health literacy':ti,ab,kw OR 'ehealth literacy':ti,ab,kw OR 'education level*':ti,ab,kw OR 'level of education':ti,ab,kw OR 'educational attainment':ti,ab,kw OR 'educational achievement':ti,ab,kw OR (((civic OR social OR community OR public) NEXT/1 (participation OR engagement OR integration OR inclusion OR involvement)):ti,ab,kw) OR 'sense of community':ti,ab,kw OR 'sense of belonging':ti,ab,kw OR isolation:ti,ab,kw OR 'social exclusion':ti,ab,kw OR loneliness:ti,ab,kw OR 'social support*':ti,ab,kw OR 'social network*':ti,ab,kw OR discrimination:ti,ab,kw OR nondiscrimination:ti,ab,kw OR 'non discrimination':ti,ab,kw OR racism:ti,ab,kw OR homophobia:ti,ab,kw OR biphobia:ti,ab,kw OR transphobia:ti,ab,kw OR 'workplace condition*':ti,ab,kw OR 'workplace environment':ti,ab,kw OR incarceration:ti,ab,kw OR imprisonment:ti,ab,kw OR prisoner*:ti,ab,kw OR 'economic stability':ti,ab,kw OR income:ti,ab,kw OR 'cost of living':ti,ab,kw OR socioeconomic*:ti,ab,kw OR 'socio-economic*':ti,ab,kw OR 'social economic status':ti,ab,kw OR ((social NEXT/1 (status OR achievement OR condition* OR protection OR polic* OR environment OR connectedness OR cohesion OR context*)):ti,ab,kw) OR 'public polic*':ti,ab,kw OR 'government* polic*':ti,ab,kw OR poverty:ti,ab,kw OR employment:ti,ab,kw OR unemployment:ti,ab,kw OR 'occupational status':ti,ab,kw OR 'job security':ti,ab,kw OR 'job insecurity':ti,ab,kw OR 'food security':ti,ab,kw OR 'food insecurity':ti,ab,kw OR housing:ti,ab,kw OR lodging*:ti,ab,kw OR 'living arrangement*':ti,ab,kw OR transportation:ti,ab,kw OR 'air quality':ti,ab,kw OR 'air pollution':ti,ab,kw OR 'water quality':ti,ab,kw OR 'healthy food*':ti,ab,kw OR 'food desert*':ti,ab,kw OR neighborhood*:ti,ab,kw OR neighbourhood*:ti,ab,kw OR 'physical environment':ti,ab,kw OR crime:ti,ab,kw OR violence:ti,ab,kw OR 'language barrier*':ti,ab,kw OR 'communication barrier*':ti,ab,kw OR 'driver* of health':ti,ab,kw OR doh:ti,ab,kw OR 'childhood development':ti,ab,kw OR 'early life development':ti,ab,kw OR 'structural conflict*':ti,ab,kw OR culture:ti,ab,kw OR stress:ti,ab,kw OR 'physical activit*':ti,ab,kw OR exercise:ti,ab,kw OR parks:ti,ab,kw OR 'green space*':ti,ab,kw OR walkability:ti,ab,kw OR 'walk friendly':ti,ab,kw OR 'walking friendly':ti,ab,kw OR playground*:ti,ab,kw) AND ('health equity'/exp OR (((equit* OR inequit* OR equal* OR inequal* OR unequal*) NEAR/3 (health* OR care)):ti,ab,kw) OR equitabl*:ti,ab,kw OR inequitabl*:ti,ab,kw) AND ('oncology'/exp OR 'cancer care'/exp OR 'cancer therapy'/exp OR oncolog*:ti,ab,kw OR cancerology:ti,ab,kw OR (((cancer OR tumor* OR tumour*) NEAR/3 (care OR treat* OR therap* OR intervention*)):ti,ab,kw) OR radiotherap*:ti,ab,kw OR chemotherap*:ti,ab,kw OR immunotherap*:ti,ab,kw)

**Cochrane Library (WileyOnline; Cochrane Database of Systematic Reviews, Cochrane Central Register of Controlled Trials, Cochrane Methodology Register) – 35** references retrieved on 15 April 2022

Using Search Manager in Advanced Search:

1. [mh "Social Determinants of Health"] or [mh "health services accessibility"] or [mh "insurance, health"] or [mh "health literacy"] or [mh "educational status"] or [mh "social participation"] or [mh "community participation"] or [mh "social inclusion"] or [mh "social integration"] or [mh "social cohesion"] or [mh "social isolation"] or [mh loneliness] or [mh "social support"] or [mh "social networking"] or [mh "social discrimination"] or [mh workplace] or [mh prisoners] or [mh "economic stability"] or [mh income] or [mh economics] or [mh "social class"] or [mh "socioeconomic factors"] or [mh "public policy"] or [mh "social environment"] or [mh poverty] or [mh employment] or [mh "food security"] or [mh "food supply"] or [mh housing] or [mh "neighborhood characteristics"] or [mh "residence characteristics"] or [mh crime] or [mh violence] or [mh transportation] or [mh "air pollution"] or [mh "water quality"] or [mh "access to healthy foods"] or [mh "food deserts"] or [mh "communication barriers"] or [mh "child development"] or [mh culture] or [mh "stress, physiological"] or [mh "stress, psychological"] or [mh exercise] or [mh "parks, recreational"]
2. ((social NEXT determin*) or (social NEXT factor*) or ("social health" NEXT determinant*) or sdoh or ("health structural" NEXT determinant*) or ((healthcare or care or "health services") NEAR/2 access*) or ((health or healthcare or "health care" or medical or sickness) NEXT (insurance or benefit*)) or "health literacy" or "ehealth literacy" or (education NEXT level*) or "level of education" or "educational attainment" or "educational achievement" or ((civic or social or community or public) NEXT (participation or engagement or integration or inclusion or involvement)) or "sense of community" or "sense of belonging" or isolation or "social exclusion" or loneliness or (social NEXT support*) or (social NEXT network*) or discrimination or nondiscrimination or "non discrimination" or racism or homophobia or biphobia or transphobia or (workplace NEXT condition*) or "workplace environment" or incarceration or imprisonment or prisoner* or "economic stability" or income or "cost of living" or socioeconomic* or socio-economic* or "social economic status" or (social NEXT (status or achievement or condition* or protection or polic* or environment or connectedness or cohesion or context*)) or (public NEXT polic*) or (government* NEXT polic*) or poverty or employment or unemployment or "occupational status" or "job security" or "job insecurity" or "food security" or "food insecurity" or housing or lodging* or (living NEXT arrangement*) or transportation or "air quality" or "air pollution" or "water quality" or (healthy NEXT food*) or (food NEXT desert*) or neighborhood* or neighbourhood* or "physical environment" or crime or violence or (language NEXT barrier*) or (communication NEXT barrier*) or (driver* NEXT "of health") or doh or "childhood development" or "early life development" or (structural NEXT conflict*) or culture or stress or (physical NEXT activit*) or exercise or parks or (green NEXT space*) or walkability or "walk friendly" or "walking friendly" or playground*):ti,ab,kw
3. #1 or #2
4. [mh "health equity"]
5. (((equit* or inequit* or equal* or inequal* or unequal*) NEAR/3 (health* or care)) or equitabl* or inequitabl*):ti,ab,kw
6. #4 or #5
7. [mh "medical oncology"] or [mh "antineoplastic protocols"] or [mh "combined modality therapy"] or [mh chemoradiotherapy] or [mh "chemotherapy, adjuvant"] or [mh "consolidation chemotherapy"] or [mh electrochemotherapy] or [mh "induction chemotherapy"] or [mh "maintenance chemotherapy"] or [mh photochemotherapy] or [mh radiotherapy] or [mh "remission induction"] or [mh ^immunotherapy]
8. (oncolog* or cancerology or ((cancer or tumor* or tumour*) NEAR/3 (care or treat* or therap* or intervention*)) or radiotherap* or chemotherap* or immunotherap*):ti,ab,kw
9. #7 or #8
10. #3 and #6 and #9

**Scopus (Elsevier) – 1087** references retrieved on 15 April 2022

One-line search run in the Advanced Search interface:

TITLE-ABS-KEY("social determin*" or "social factor*" or "social health determinant*" or sdoh or "health structural determinant*" or ((healthcare or care or "health services") W/2 access*) or ((health or healthcare or "health care" or medical or sickness) PRE/0 (insurance or benefit*)) or "health literacy" or "ehealth literacy" or "education level*" or "level of education" or "educational attainment" or "educational achievement" or ((civic or social or community or public) PRE/0 (participation or engagement or integration or inclusion or involvement)) or "sense of community" or "sense of belonging" or isolation or "social exclusion" or loneliness or "social support*" or "social network*" or discrimination or nondiscrimination or "non discrimination" or racism or homophobia or biphobia or transphobia or "workplace condition*" or "workplace environment" or incarceration or imprisonment or prisoner* or "economic stability" or income or "cost of living" or socioeconomic* or socio-economic* or "social economic status" or (social PRE/0 (status or achievement or condition* or protection or polic* or environment or connectedness or cohesion or context*)) or "public polic*" or (government* PRE/0 polic*) or poverty or employment or unemployment or "occupational status" or "job security" or "job insecurity" or "food security" or "food insecurity" or housing or lodging* or "living arrangement*" or transportation or "air quality" or "air pollution" or "water quality" or "healthy food*" or "food desert*" or neighborhood* or neighbourhood* or "physical environment" or crime or violence or "language barrier*" or "communication barrier*" or (driver* PRE/0 "of health") or doh or "childhood development" or "early life development" or "structural conflict*" or culture or stress or "physical activit*" or exercise or parks or "green space*" or walkability or "walk friendly" or "walking friendly" or playground*) AND TITLE-ABS-KEY(((equit* or inequit* or equal* or inequal* or unequal*) W/3 (health* or care)) or equitabl* or inequitabl*) AND TITLE-ABS-KEY(oncolog* or cancerology or ((cancer or tumor* or tumour*) W/3 (care or treat* or therap* or intervention*)) or radiotherap* or chemotherap* or immunotherap*)

**Dissertations & Theses Global (ProQuest) – 59** references retrieved on 15 April 2022

One-line search run in command line interface of Advanced Search:

AB,TI("social determin*" or "social factor*" or "social health determinant*" or sdoh or "health structural determinant*" or ((healthcare or care or "health services") NEAR/2 access*) or ((health or healthcare or "health care" or medical or sickness) PRE/0 (insurance or benefit*)) or "health literacy" or "ehealth literacy" or "education level*" or "level of education" or "educational attainment" or "educational achievement" or ((civic or social or community or public) PRE/0 (participation or engagement or integration or inclusion or involvement)) or "sense of community" or "sense of belonging" or isolation or "social exclusion" or loneliness or "social support*" or "social network*" or discrimination or nondiscrimination or "non discrimination" or racism or homophobia or biphobia or transphobia or "workplace condition*" or "workplace environment" or incarceration or imprisonment or prisoner* or "economic stability" or income or "cost of living" or socioeconomic* or socio-economic* or "social economic status" or (social PRE/0 (status or achievement or condition* or protection or polic* or environment or connectedness or cohesion or context*)) or "public polic*" or (government* PRE/0 polic*) or poverty or employment or unemployment or "occupational status" or "job security" or "job insecurity" or "food security" or "food insecurity" or housing or lodging* or "living arrangement*" or transportation or "air quality" or "air pollution" or "water quality" or "healthy food*" or "food desert*" or neighborhood* or neighbourhood* or "physical environment" or crime or violence or "language barrier*" or "communication barrier*" or (driver* PRE/0 "of health") or doh or "childhood development" or "early life development" or "structural conflict*" or culture or stress or "physical activit*" or exercise or parks or "green space*" or walkability or "walk friendly" or "walking friendly" or playground*) AND AB,TI(((equit* or inequit* or equal* or inequal* or unequal*) NEAR/3 (health* or care)) or equitabl* or inequitabl*) AND AB,TI(oncolog* or cancerology or ((cancer or tumor* or tumour*) NEAR/3 (care or treat* or therap* or intervention*)) or radiotherap* or chemotherap* or immunotherap*)

**Trip Database Pro – 850** references retrieved on 19 March 2022

("language barrier" OR "communication barrier" OR "drivers of health" OR doh OR "childhood development" OR "early life development" OR "structural conflict" OR culture OR stress OR "physical activity" OR "physical activities" OR exercise OR parks OR "green space" OR "green spaces" OR walkability OR "walk friendly" OR "walking friendly" OR playground* OR employment OR unemployment OR "occupational status" OR "job security" OR "job insecurity" OR "food security" OR "food insecurity" OR housing OR lodging OR "living arrangement" OR transportation OR "air quality" OR "air pollution" OR "water quality" OR "healthy food" OR "healthy foods" OR "food desert" OR neighborhood* OR neighbourhood* OR "physical environment" OR crime OR violence OR "workplace conditions" OR "workplace environment" OR incarceration OR imprisonment OR prisoner* OR "economic stability" OR income OR "cost of living" OR "socioeconomic status" OR "social status" OR "social condition" OR "social protection" OR "social environment" OR "social cohesion" OR "social connectedness" OR "social context" OR "public policy" OR "public policies" OR "social policy" OR "social policies" OR "government policy" OR "governmental policy" OR poverty OR "social determinant" OR "social determinants" OR sdoh OR "healthcare access" OR "care access" OR "access to care" OR "health insurance" OR "medical insurance" OR "health literacy" OR "ehealth literacy" OR "education level" OR "level of education" OR "educational attainment" OR "educational achievement" OR "civic participation" OR "civic engagement" OR "community engagement" OR "community participation" OR "community involvement" OR "social participation" OR "public participation" OR "social inclusion" OR "social integration" OR "sense of community" OR "sense of belonging" OR isolation OR "social exclusion" OR loneliness OR "social support" OR "social network" OR discrimination OR nondiscrimination OR "non discrimination" OR racism OR homophobia OR biphobia OR transphobia) AND ("health equity" OR equitabl* OR inequitabl* OR "healthcare equity" OR "care equity" OR "healthcare inequity" OR "care inequity") AND (oncolog* OR cancerology OR "cancer care" OR "cancer treatment" OR "cancer therapy" OR "cancer therapies" OR "cancer interventions" OR chemotherap* OR radiotherap* OR immunotherap*)

**American Cancer Society – 22** results on 13 October 2022

Search for “social determinants of health”

<https://www.cancer.org/search.html?q=%22social+determinants+of+health%22>

**American Society of Clinical Oncology – 85** results on 13 October 2022

Search for “social determinants of health equity”, with funding opportunities, sessions, journals, abstracts/presentations, and patient resources categories removed

<https://beta.asco.org/search?q=social%20determinants%20of%20health%20equity&filters=%7B%22contentTypeGroupLabel%22:%5B%7B%22key%22:%22General%20Information%22%7D,%7B%22key%22:%22News%22%7D,%7B%22key%22:%22Member%20Blogs%22%7D,%7B%22key%22:%22Guidelines%22%7D,%7B%22key%22:%22ASCO%20Educational%20Book%22%7D%5D%7D&groupBy=CONTENT_TYPE>

**Association of Community Cancer Centers – 63** results on 24 October 2022

Search for “social determinants of health” AND equity

<https://www.accc-cancer.org/search-results?Keywords=%22social%20determinants%20of%20health%22%20AND%20equity>

**Community Oncology Alliance – 60** results on 3 February 2023

Search for “social determinants of health” – 10 results

<https://communityoncology.org/?s=%22social+determinants+of+health%22>

Search for “health equity” – 50 results

<https://communityoncology.org/?s=%22health+equity%22>

**Cancer Support Community – 45** results on 17 October 2022

Search for “health equity” – 38 results

<https://www.cancersupportcommunity.org/search?search=+%22health+equity%22>

Search for “social determinants” – 7 results

<https://www.cancersupportcommunity.org/search?search=%22social+determinants%22>

**Optum – 610** results on 31 October 2022

Search for social determinants of health equity, review Insights and News categories only

<https://www.optum.com/search-results.html?query=+social+determinants+of+health+equity&referrerPageUrl=https://www.optum.com/>
