# Supplementary material for: Which social determinants of health have the highest impact in community oncology to advance patient care equity and improve health outcomes? A scoping review
Source: Cancer Med. 2024 Sep 6;13(17):e70160. doi: 10.1002/cam4.70160 (PMC11378356; doi:10.1002/cam4.70160)
Supplement: Supplementary file 2 — Figure S1. PRISMA diagram of selection process for included website records in scoping review. [file CAM4-13-e70160-s004.docx]

**Supplementary Figure 1. PRISMA diagram of selection process for included website records in scoping review.**

**Identification of records via websites**

Records identified from websites (n = 885):

American Society of Clinical Oncology (n = 85)

Association of Community Cancer Centers (n = 63)

Cancer Support Community (n = 45)

American Cancer Society (n = 22)

Optum Life Sciences (n = 610)

Community Oncology Alliance (n = 60)

**Identification**

Records excluded (n = 861):

American Society of Clinical Oncology (n = 76)

Association of Community Cancer Centers (n = 53)

Cancer Support Community (n = 44)

American Cancer Society (n = 21)

Optum Life Sciences (n= 610)

Community Oncology Alliance (n = 57)

Total records screened

(n = 885)

**Screening**

Records included in review (n = 24):

American Society of Clinical Oncology (n = 9)

Association of Community Cancer Centers (n = 10)

Cancer Support Community (n = 1)

American Cancer Society (n = 1)

Optum Life Sciences (n = 0)

Community Oncology Alliance (n = 3)

**Included**
