# Supplementary material for: Which social determinants of health have the highest impact in community oncology to advance patient care equity and improve health outcomes? A scoping review
Source: Cancer Med. 2024 Sep 6;13(17):e70160. doi: 10.1002/cam4.70160 (PMC11378356; doi:10.1002/cam4.70160)
Supplement: Supplementary file 3 — Table S1. Cancer Types Reported (n = 78). [file CAM4-13-e70160-s002.docx]

| **Supplementary Table 1. Cancer Types Reported (n=78)** | |
| --- | --- |
| **Cancer Type** | **Frequency** |
| Central Nervous System (CNS) | 2 |
| Breast | 33 |
| Gastrointestinal (GI) | 24 |
| Genitourinary (GU) | 19 |
| Gynecological (GYN) | 14 |
| Head and Neck | 6 |
| Hematologic (HEM) | 8 |
| Lung/Thoracic | 10 |
| Skin | 4 |
| Undisclosed* | 21 |
| *Undisclosed: Text did not delineate which cancer(s) were evaluated  CNS includes brain and medulloblastoma  GI includes colon/colorectal, gastrointestinal, liver, pancreatic, stomach  GU includes the bladder, genitourinary, kidney, prostate, testicular, urothelial  GYN includes cervical, gynecological, ovarian, uterus, vulva/vaginal  Head and neck include head and neck, oral, thyroid  HEM includes hematologic, hematopoietic, leukemia, non-Hodgkin lymphoma  Skin includes non-melanoma | |
|  | |
