# Supplementary material for: Which social determinants of health have the highest impact in community oncology to advance patient care equity and improve health outcomes? A scoping review
Source: Cancer Med. 2024 Sep 6;13(17):e70160. doi: 10.1002/cam4.70160 (PMC11378356; doi:10.1002/cam4.70160)
Supplement: Supplementary file 4 — Table S2. Extended Results: Confirmatory Studies Addressing SDOH and Assessing the Impact on Health Equity (n = 17). [file CAM4-13-e70160-s001.docx]

| **Supplementary Table 2. Extended Results: Confirmatory Studies Addressing SDOH and Assessing the Impact on Health Equity (n=17)** | | | | | | | |
| --- | --- | --- | --- | --- | --- | --- | --- |
| **Author (year)** | **Study Design** | **Cancer Type** | **Geography/Population Characteristics** | **SDOH / Health Equity Pair** | | **Impact** | **Conclusions** |
| Adams  (1996)^29^ | Cross sec | CRC | Texas, Urban; Black/African American, Female and Male, Aged 35-66 years old | Hlth/gen lit-pat | Adh/ comp | NC | A health information intervention presented by a registered professional nurse (direct teaching and interaction) has no significant bearing on subjects' health practices, e.g., intent to obtain annual screening for colorectal cancer compared to subjects who were given relevant, culturally sensitive, informative pamphlets with a low reading level to read on their own. |
| Carthon (2021)^37^ | Sys Rev | PC | Undisclosed geography; Black/African American and White, Male | Education | Adh/ comp | + | Community education can improve Black men’s knowledge of prostate cancer, increase awareness of resources, and improve shared decision-making about screening. It is essential to engage Black men in culturally appropriate and community-specific initiatives to address screening and treatment disparities; communities’ preferences for format and methods of delivering education (in-person interactive sessions, videos, lecture instead of print-based educational material), location (barber shops and churches). |
|  |  |  |  | Education | ATC/ Tx | + |  |
| Chavarria (2021)^38^ | Qual; RCT | BC, CRC | Undisclosed geography; Black/African American, White, and Other including Hispanic, Latino, or Spanish origin, Female and Male | Hlth/gen lit-pat | ATC/ Tx | Info | Adaptation of a brochure to raise awareness about inherited breast cancer via learner verification and revision method identified areas for modification: attraction (color scheme to represent the African American flag, photos to appeal to entire target audience); comprehension (terms such as “hereditary” substituted with “runs in the family”); cultural-linguistic acceptability (preferred term “women of color”). |
|  |  |  |  | Soc incl & non-discrim | Adh/ comp | NC | The Latinos CARES intervention (photo novella and video based on learner verification and revision) was piloted (n=76) to promote FIT uptake. The intervention group, who received the “transcreated” health materials, demonstrated slightly higher FIT uptake (90%) than did the comparison group (83%); not statistically significant. |
| Gil  (2016)^39^ | Descrip | ALL, MEL, MB | Undisclosed geography; Hispanic, Latino, or Spanish origin | Hlth/gen lit-pat | Adh/ comp | Info | Limited English Proficiency (LEP) Patient Family Advocates serve as a specialized medical interpreter, cultural liaison, and healthcare systems advocate/adjunct case manager to promote continuity of care, decrease stress and burden, enhance patient safety, foster trust and connectedness, aid communication, demonstrate respect for patients and families. It became clear that there were gaps and barriers beyond language standing in the way of effective communication and treatment adherence. A language-concordant team member allows easier access to important resources by coordination of care (psychologist, social worker, medical issues). The LEP Advocate enhanced the safety of complex chemotherapy treatment and created safer systems for families caring for their child at home, such as a medication calendar system available in both higher literacy (word-based) and lower literacy (color-coded) versions. |
|  |  |  |  | Soc incl & non-discrim | Adh/ comp | Info |  |
|  |  |  |  | Soc incl & non-discrim | ATPM | Info |  |
| Holle (2020)^40^ | Descrip | CRC | Connecticut, Urban; Black/African American and White including Hispanic, Latino, or Spanish origin, Minimum age 45 years old | Access afford hlth srv | Adh/ comp | Info | The community pharmacist certified in CRC can facilitate CRC risk education, identify patients who are eligible for screening, provide them with a FIT test, and then support patients so that they complete and send in their test for analysis. Participants rated “great” satisfaction with the service. The follow-through among these socioeconomically disadvantaged patients to use and turn in the FIT testing kit shows that this is a viable way to engage hard to reach eligible patients in the community. Some aspects of the program participants reported they liked included: pharmacist’s listening skills and ability to explain concepts and answer questions, the quality of advice provided, the level of friendliness and helpfulness. |
|  |  |  |  | Hlth/gen lit-pat | Adh/ comp | Info |  |
|  |  |  |  | Income & soc prtctn | Adh/ comp | Info |  |
|  |  |  |  | Prov comm & cultrl comp | ATC/ Tx | Info |  |
| Kim (2016)^22^ | Sys Rev | CC, BC, CRC, OC | United States, Canada, India, Pakistan, Taiwan; Asian, Black/African American, White including Hispanic, Latino, or Spanish origin, Female and Male, Aged 32-71 years old | Income & soc prtctn | ATC/ Tx | Info | Community based healthcare workers (CBHWs) are typically trusted members of their communities with whom they share the same cultural and linguistic backgrounds and life experiences. They are positioned to provide tailored, culturally responsive interventions. CBHWs as an intervention model can be cost-effective for certain conditions/behaviors for low-income, underserved, and racial and ethnic minority communities. CBHWs promote more health services such as cancer screenings and patient navigation. |
|  |  |  |  | Soc incl & non-discrim | ATC/ Tx | Info |  |
| Meade (2020)^41^ | Lit Rev | U | Undisclosed geography and population characteristics | Prov comm & cultrl comp | Adh/ comp | Info | Effective communication influence rates of emotional and physiological health, adherence to treatment regimens, decision-making about treatment options, acceptance of recommended treatment including chemotherapy, end-of-life preparation, and patient satisfaction and overall enhanced quality of life. It has been observed that many older cancer patients experience worse patient-provider communication than their younger counterparts. Differences in the quality of patient-provider communication (e.g., respecting, listening, explaining, and time spent with the patient) have been noted based upon race/ethnicity. These reports underscore the importance of developing strong patient-provider relationships through culturally competent communication. |
|  |  |  |  | Prov comm & cultrl comp | ATC/ Tx | Info |  |
|  |  |  |  | Soc incl & non-discrim | Adh/ comp | Info |  |
|  |  |  |  | Soc incl & non-discrim | ATC/ Tx | Info |  |
|  |  |  |  | Soc incl & non-discrim | QOL | Info |  |
| Okoro (2020)^42^ | Qual | U | Minnesota, Urban; Black/African American and Other, Female and Male, Aged 18-86 years old | Education | Adh/ comp | Info | The community-engaged educational intervention (didactic and interactive, eight 90-min sessions) aimed to enhance the health of African American (AA) men in a geographical area that lacks the social institutions commonly associated with the Black/AA community such as Black churches and barbershops. Leveraging social networks characteristic of the AA culture and involving women within those networks, may enhance the health of AA men. Participants became more knowledgeable/aware of the risk factors and treatment of conditions. Female participants shared with males in their family/social networks. Participants were unaware of how to initiate processes in the health-care system or advocate for health-care needs. Male participants reported: empowerment to take a more active role in their health care (requesting appointments/tests, asking the provider clarifying questions); lifestyle changes (dietary habits and increase in physical activity); feeling healthier; the role women played in keeping them accountable to a healthier lifestyle. |
|  |  |  |  | Education | ATC/ Tx | Info |  |
|  |  |  |  | Education | QOL | Info |  |
|  |  |  |  | Hlth/gen lit-pat | ATC/ Tx | Info |  |
|  |  |  |  | House, amenities, environ | QOL | Info |  |
| Otero* (2022)^43^ | Qual | PC, PaC | Undisclosed geography; Black/African American and Other including Hispanic, Latino, or Spanish origin | Hlth/gen lit-pat | Adh/ comp | Info | Hosting community outreach events via social media platforms to increase awareness/knowledge of prostate and pancreatic cancer screening, treatment options, research, and causes and solutions to disparities in the Black and Latinx community via “bed to bedside” model allowed participants to gain a well-rounded understanding of the cancer process from cancer survivors, physicians, and researchers of color. Conducting outreach activities via these platforms eliminated transportation barriers. Participants reported they enjoyed the “bed to bedside” model; hearing from a survivor of color led them feeling more comfortable in considering cancer screening. |
|  |  |  |  | House, amenities, environ | ATC/ Tx | Info |  |
|  |  |  |  | Education | Adh/ comp | Info |  |
|  |  |  |  | Preparing for care | ATC/ Tx | Info |  |
|  |  |  |  | Soc incl & non-discrim | Adh/ comp | Info |  |
| Patel (2020)^44^ | RCT | LC, BC, GI, GU, HEM, U | New Jersey, Illinois, Urban; Asian, Black/African American, Native Hawaiian or Other Pacific Islander, White, Female and Male, Aged at least 18 years old | Income & soc prtctn | ATC/ Tx | Info | This multi-level intervention uses a lay health worker to improve care delivery for low-income and minority patients after a diagnosis of cancer through education as well as advance care planning and symptom management with their providers. The design will evaluate the impact on patient-reported outcomes, primarily on the quality of life, patient activation, symptom burden, healthcare use, and costs. The study will provide crucial information regarding the feasibility of such an approach to inform future scale and spread of the “Lay health worker Educates Engages and Activates Patients to Share (LEAPS)” intervention among other patient populations. |
|  |  |  |  | Soc incl & non-discrim | ATC/ Tx | Info |  |
|  |  |  |  | Access afford hlth srv | QOL | Info |  |
| Patel* (2021)^30^ | RCT | BC, LC, U | New Jersey; Black/African American, White, and Other, Female and Male | Income & soc prtctn | ATC/ Tx | + | Integration of community-based interventions into cancer care for low-income and minority populations may be a more effective and sustainable way to ensure equitable cancer care. The intervention uses community health workers trained to activate patients to discuss advance care planning and their symptom burden with cancer clinicians and to connect patients with culturally-relevant community resources to overcome complications from social determinants of health. At 4 months, the intervention group had greater improvements in quality of life as compared to the control group, greater change in patient activation, and lower acute care use (hospital visits and emergency department use). |
|  |  |  |  | Income & soc prtctn | QOL | + |  |
|  |  |  |  | Preparing for care | ATC/ Tx | + |  |
|  |  |  |  | Preparing for care | QOL | + |  |
|  |  |  |  | Soc incl & non-discrim | QOL | + |  |
|  | | | | | | | |
| Percac-Lima (2014)^31^ | Cohort | BC | Massachusetts, Urban; Asian, Black/African American, White, and Other including Hispanic, Latino, or Spanish origin, Female | Access afford hlth srv | TTNT | + | Patient navigation (PN) improved appropriate follow-up of the abnormal mammograms by 15% compared to the non-navigated group. The findings of this study suggest that among disadvantaged women, PN increases appropriate follow-up care after an abnormal screening mammogram. To improve equity and quality of cancer care, PN should be expanded to include high-risk disadvantaged patients within primary care networks. |
| Rariy* (2021)^32^ | Cross sec | U | Rural; Undisclosed population characteristics | Access afford hlth srv | TTNT | + | Telehealth provides a bridge to the rural health access gap, and advances health equity by offering specialized care to patients in their local communities. A 100% retention rate for all 14 patients that were enrolled, the turnaround time from the treatment plan to treatment was 6 business days, and a total of 62 oncology telehealth appointments reducing patient travel by a total of 12,705 miles, 310 travel hours, and saving $7,380 in travel costs and accommodation. |
|  |  |  |  | House, amenities, environ | ATC/ Tx | + |  |
|  |  |  |  | House, amenities, environ | TTNT | + |  |
| Reynolds* (2020)^33^ | Cohort | U | Undisclosed geography; Asian, Black/African American, and Other including Hispanic, Latino, or Spanish origin | Soc incl & non-discrim | ATPM | + | Partnerships with several community cancer providers led to the successful enrollment of almost 400 underrepresented minority (URM) patients into the study, which seeks to identify the molecular drivers of a patient’s cancer. Providing access to tumor molecular profiling is a necessary first step to addressing cancer disparities experiences by URMs. Two hundred fifty-six patients have had samples successfully sequenced, 43% of which had at least one actionable alteration identified. Additionally, 14 patients have had positive germline results, revealing mutations that were not identified prior to their participation. |
|  | | | | | | | |
| Smith (2016)^34^ | Descrip | BC | Illinois, Urban; Black/African American, Female | Hlth/gen lit-pat | ATC/ Tx | Info | The pilot, Sisters Saving Lives, implemented training for African American (AA) breast cancer survivors demonstrated a positive impact on knowledge of breast health basics using a multifaceted and culturally sensitive approach. The trained ambassadors were confident and comfortable delivering the educational intervention and learned strategies to establish relationships with key community members. Training AA breast cancer survivors to deliver breast health education to underserved populations can help increase awareness about breast cancer and screenings. |
|  |  |  |  | Hlth/gen lit-pat | Adh/ comp | Info |  |
|  |  |  |  | Soc incl & non-discrim | Adh/ comp | Info |  |
| Strom* (2017)^35^ | Descrip | BC, GI, HEM, Thorac | North Carolina; Black/African American and Other including Hispanic, Latino, or Spanish origin, Female and Male | Income & soc prtctn | ATC/ Tx | + | Cancer Services Inc. plays an important role in facilitating the procurement of much-needed cancer care supplies and services that are not paid for by insurance; predominately  medication. Such support alleviates the financial toxicity of cancer care, which according to a recent study reported in Cancer, affects 29% of cancer survivors, the majority of whom (86%) have health insurance. |
|  | | | | | | | |
| Thompson (2018)^36^ | Lit Rev | BC | Illinois, Ohio, Washington; Black/African American and White including Hispanic, Latino, or Spanish origin, Female | Access afford hlth srv | Death | Info | Women of color, socially and economically disadvantaged women, and women who live in rural areas suffer from increased mortality after diagnosis.  Three case studies noted working with communities at a variety of levels of influence; education, arranging treatment, or working with a community’s resources. These three studies used a “meeting the women where they are” strategy.  Education can help women overcome the intrapersonal and interpersonal barriers to screening, including cultural barriers.  Mammovans/assistance in traveling to screening facilities can reduce some of the geographic barriers.  Patient navigators (PNs) can assist women through the process of both screening and treatment by: identifying financial support for mammograms and scheduling a convenient appointment, addressing barriers to screening, reminder calls, and following-up with test results, including resolution of abnormalities and assurance of treatment.  The use of promotores (bilingual and bicultural lay health workers from the community), to educate Latinas about breast cancer resulted in improved knowledge about breast cancer, increased report of discussions with doctors regarding mammograms, and increased intention to obtain a mammogram. Promotores travel to study participants’ homes, where they facilitate home health parties (HHPs). |
|  |  |  |  | Education | ATC/ Tx | + |  |
|  |  |  |  | Income &  soc prtctn | ATC/ Tx | + |  |
|  |  |  |  | House, amenities, environ | ATC/ Tx | + |  |
|  |  |  |  | Soc incl & non-discrim | ATC/ Tx | + |  |

**Note:** Results are based upon confirmatory (i.e., intervention) studies addressing social determinants of health (SDOH) and assessing the impact on health equity measures. Results from exploratory studies identifying SDOH and health equity issues were not included.

*Abstract only

**Study Design:** Cross sec - Cross sectional; Descrip - Descriptive; Lit Rev - Literature Review; Qual - Qualitative research; RCT - Randomized controlled trial; Sys Rev - Systematic review

**Type of Cancer:** ALL - Acute lymphoblastic leukemia; BC - Breast cancer; CC - Cervical cancer; CRC - Colorectal cancer; GI - Gastrointestinal; GU - Genitourinary; HEM - Hematologic; LC - Lung cancer; MB - Medulloblastoma; MEL - Melanoma; OC - Oral cancer; PaC - Pancreatic cancer; PC - Prostate cancer; Thorac - Thoracic; U - Undisclosed

**SDOH:** Access afford hlth srv - Access to affordable health services of decent quality; House, amenities, environ - Housing, basic amenities and the environment; Hlth/gen lit- pat - Health/general literacy-patient; Income & soc prtctn - Income and social protection; Prep for care - Preparing for care; Pat-provider comm - Pat-provider communication; Soc incl & non-discrim - Social inclusion and non-discrimination

**Health Equity:** Adh/comp - Adherence/compliance; ATC/Tx - Access to care/treatment; ATPM - Access to precision medicine; QOL - Quality of life

**Impact:** Info (Informational); NC (No change); + (Positive)
